# Co-infection of Ticks: The Rule Rather Than the Exception

**DOI:** 10.1371/journal.pntd.0004539

**Published:** 2016-03-17

**Authors:** Sara Moutailler, Claire Valiente Moro, Elise Vaumourin, Lorraine Michelet, Florence Hélène Tran, Elodie Devillers, Jean-François Cosson, Patrick Gasqui, Van Tran Van, Patrick Mavingui, Gwenaël Vourc’h, Muriel Vayssier-Taussat

**Affiliations:** 1 UMR Bipar, Anses, INRA, ENVA 14 Rue Pierre et Marie Curie, Maisons-Alfort, France; 2 Université de Lyon, Lyon, France; Université Lyon 1, Villeurbanne, France; CNRS, UMR5557, Ecologie Microbienne, Villeurbanne, France; INRA, UMR1418, Villeurbanne, France; 3 EPIA, INRA, 63122 Saint Genès Champanelle, France; 4 CBGP, INRA, Vetagrosup, IRD F-34988 Montferrier-sur-Lez, France; 5 Université de La Réunion, UMR PIMIT, INSERM 1187, CNRS 9192, IRD 249, Plateforme de Recherche CYROI, Saint-Denis, La Réunion, France; University of California San Diego School of Medicine, UNITED STATES

## Abstract

**Introduction:**

Ticks are the most common arthropod vectors of both human and animal diseases in Europe, and the *Ixodes ricinus* tick species is able to transmit a large number of bacteria, viruses and parasites. Ticks may also be co-infected with several pathogens, with a subsequent high likelihood of co-transmission to humans or animals. However few data exist regarding co-infection prevalences, and these studies only focus on certain well-known pathogens. In addition to pathogens, ticks also carry symbionts that may play important roles in tick biology, and could interfere with pathogen maintenance and transmission. In this study we evaluated the prevalence of 38 pathogens and four symbionts and their co-infection levels as well as possible interactions between pathogens, or between pathogens and symbionts.

**Methodology/principal findings:**

A total of 267 *Ixodes ricinus* female specimens were collected in the French Ardennes and analyzed by high-throughput real-time PCR for the presence of 37 pathogens (bacteria and parasites), by rRT-PCR to detect the presence of Tick-Borne encephalitis virus (TBEV) and by nested PCR to detect four symbionts. Possible multipartite interactions between pathogens, or between pathogens and symbionts were statistically evaluated. Among the infected ticks, 45% were co-infected, and carried up to five different pathogens. When adding symbiont prevalences, all ticks were infected by at least one microorganism, and up to eight microorganisms were identified in the same tick. When considering possible interactions between pathogens, the results suggested a strong association between *Borrelia garinii* and *B*. *afzelii*, whereas there were no significant interactions between symbionts and pathogens.

**Conclusion/significance:**

Our study reveals high pathogen co-infection rates in ticks, raising questions about possible co-transmission of these agents to humans or animals, and their consequences to human and animal health. We also demonstrated high prevalence rates of symbionts co-existing with pathogens, opening new avenues of enquiry regarding their effects on pathogen transmission and vector competence.

## Author Summary

Ticks transmit more pathogens than any other arthropod, and one single species can transmit a large variety of bacteria and parasites. Because co-infection might be much more common than previously thought, we evaluated the prevalence of 38 known or neglected tick-borne pathogens in *Ixodes ricinus* ticks. Our results demonstrated that co-infection occurred in almost half of the infected ticks, and that ticks could be infected with up to five pathogens. Moreover, as it is well established that symbionts can affect pathogen transmission in arthropods, we also evaluated the prevalence of four symbiont species and demonstrated that all ticks were infected by at least one microorganism. This work highlights the co-infection phenomenon in ticks, which may have important implications for human and animal health, emphasizing the need for new diagnostic tests better adapted to tick-borne diseases. Finally, the high co-occurrence of symbionts and pathogens in ticks, reveals the necessity to also account for these interactions in the development of new alternative strategies to control ticks and tick-borne disease.

## Introduction

Ticks are the most common arthropod vectors of disease agents to humans and domestic animals in Europe [1]. *Ixodes ricinus* is the most important tick in terms of human and animal health as it can attach to vertebrate hosts for up to ten days, and takes a blood meal during each of its three life stages of larva, nymph and adult, except adult males who mate with feeding adult females. The natural hosts of *I*. *ricinus* include almost all wild or domestic animals living in woods and pasture, whereas humans become accidental hosts when entering tick habitats. Of all ticks, *I*. *ricinus* transmits the greatest variety of pathogens i.e., microorganisms able to cause disease in animals or humans [2]. This is likely due to the large variety of animals from which they can ingest blood, exposing the ticks to any pathogens currently infecting the hosts, including bacteria (*Borrelia burgdorferi* sensu lato group: *Borrelia burgdorferi* sensu stricto, *B*. *garinii*, *B*. *afzelii*, *B*. *spielmanii* [3], *Anaplasma phagocytophilum*, the spotted fever group of *Rickettsia* sp., and possibly *Bartonella* spp. and *Candidatus* Neoehrlichia mikurensis…), parasites (mainly *Babesia*), and viruses (mainly tick-borne encephalitis virus). Questing larvae can only be infected by those few pathogens capable of horizontal transmission in ticks. Thus larvae have a limited potential role as vectors of human or animal pathogens. In contrast, the importance of nymphs on the impact of tick-borne pathogens on public health is readily recognized. Indeed, questing nymphs have already been exposed to pathogens during their blood meal as larvae. They are often able to transmit pathogens, especially considering nymph bites often go unnoticed due to their tiny size, and that transmission rates increase with lengthening meal duration [4]. Questing adults can also bite humans, and are the cause of between 10 to 40% of all human tick bites in Europe [5–8]. During the larval and nymphal stages they have ingested two blood meals, both potential pathogen-acquiring occasions. Thus, they are more likely to be infected and co-infected than nymphs [7,9,10] and represent a substantial threat to the public health, especially in term of co-infections. However, they are less numerous in the environment [11] and they are often removed earlier than nymphs because they are more easily detected.

Ticks co-infection [12–18], and co-transmission of pathogens [19–28] might have important potential implications and hence be highly relevant to public health [29]. Indeed co-infection in humans and animals might enhance disease severity as has been reported for concurrent babesiosis and Lyme disease [30,31], and may also have significant consequences in terms of tick-borne disease treatment and diagnosis [29].

In addition to human and animal pathogens, ticks also carry symbionts (any interacting species [32]) that may not only play a role in tick biology, but might also interact with pathogens [33]. Occasionally, the distinction between pathogens and endosymbionts is blurred. For instance, some authors state that *Rickettsia* species are primarily endosymbionts that are vertically transmitted by arthropods, and only exist secondarily as vertebrate pathogens [34]. Secondly, *Coxiella burnetii* is mostly reported as a vertebrate pathogen, even though numerous other *Coxiella* species have been identified in ticks. In fact, it was recently demonstrated that the inherited tick symbiont *C*. *burnetii* has emerged and is now able to infect vertebrates [35]. Thirdly, *Wolbachia* is a common symbiont widespread in insects affecting the reproduction and/or immunity of their hosts [36] [37]. This species has also been found associated with ticks [38]. A recent experimental approach has revealed that the presence of *Wolbachia* in *I*. *ricinus* was due to parasitism from the parasitoid wasp, *Ixodiphagus hookeri* [39]. And finally, the *I*. *ricinus* endosymbiont *Midichloria mitochondrii* is detected in nearly all *I*. *ricinus* females derived from natural populations [40]. The high prevalence and transovarial transmission of this symbiont suggest that an obligate association exists, playing a crucial role in tick fitness. However, in laboratory-raised *I*. *ricinus* colonies, its prevalence decreased, indicating that any advantage acquired by the tick may only be evident under natural conditions [41]. *M*. *mitochondrii* has also recently been reconsidered as a potential vertebrate pathogen [42].

Besides their likely important roles in tick biology, tick symbionts may also interfere with pathogen transmission. For instance, endosymbionts belonging to the rickettsial genera are thought to alter transmission of other rickettsial pathogens, as seen by the inverse relationship between the infection prevalence of *R*. *rickettsii* (pathogen) and *R*. *peacockii* (symbiont) in *Dermancentor andersoni* [38,43]. Furthermore, the presence of *Coxiella*-related symbionts in the salivary glands of *Amblyomma* ticks impairs transmission of *Ehrlichia chaffeensis* [44]. In addition to symbionts, ticks are also colonized with naturally-occurring bacterial microbiota, mainly belonging to the Proteobacteria, Firmicutes, and Bacteroides phyla [45,46]. Tick microbiomes can also interfere with pathogens. As an example, ticks bred in a sterile environment exist without a normal microbiota, this alters their gut integrity and modifies *B*. *burgdorferi*’s ability to colonize this niche [45]. Microbiome alterations may also result in modulated immune responses, which might then interfere with pathogen survival and infection, as demonstrated for other arthropod vectors [47].

Until now, most studies addressing tick co-infection have only been able to assess limited numbers of pathogens at a time, such that they are unable to generate a clear and an accurate representation of all pathogens present in ticks. To overcome this limitation, we have developed a novel high-throughput method to identify both major and neglected European tick-borne pathogens (bacteria and parasites), representing up to 37 different species of bacteria and parasites, in a single sample [48]. In this study, we evaluated the prevalence of these 37 different known and neglected bacterial and parasitic tick-borne pathogens, and together with TBEV, determined the co-infection level, and any possible interactions between the detected pathogens in adult females. Females were analyzed as they have had an additional blood meal compared to nymphs, and thus are more likely to be infected and co-infected thereby increasing chances of identifying co-infections and potential interactions. As symbionts might have significant effects on both pathogens (replication, survival, etc.) and ticks, we also determined the presence of four suspected or known bacterial tick symbionts in parallel. i.e., *Wolbachia* sp., *Midichloria mitochondrii*, S*piroplasma* spp. and *Acinetobacter* spp. (as common gut inhabitants of many arthropod species), and assessed possible multipartite interactions.

## Materials and Methods

### Ticks

**Fig 1:**
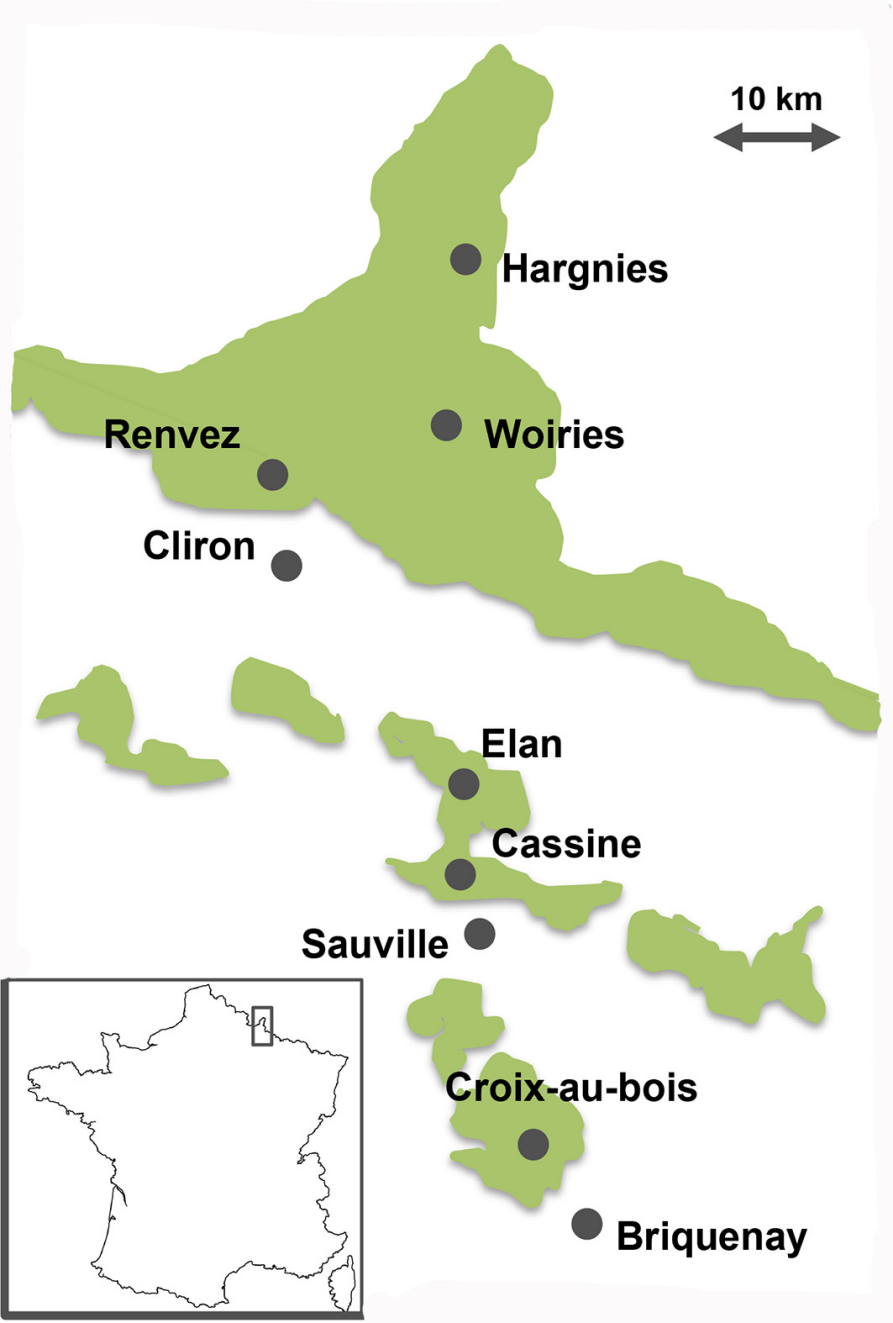
Map of the Ardennes region (France) showing the 9 tick sampling sites. Elements colored in green correspond to large forested areas.

From May to August 2012, 267 questing *Ixodes ricinus* female ticks were collected by flagging along a transect line of approximately 80 km in the French Ardennes, a region endemic for rodent-borne hantaviruses, during the course of a *Puumala* hantavirus epidemiological study (Fig 1, [49]). Along this transect, we sampled six forested sites and three sites with fragmented habitats (i.e. hedge networks). All collected ticks were surface sterilized and individually crushed as previously described [50].

### RNA and genomic DNA extraction

Genomic tick DNA was extracted from 100 μL of crushed tick using the Wizard genomic DNA purification kit (Promega, France) according to manufacturer’s instructions. RNA was extracted from 100 μL of crushed tick using the Nucleospin RNA II kit (Macherey Nagel, Germany) according to the manufacturer’s instructions. Purified DNA and RNA were eluted into 50 μL of either elution buffer or RNase-free water respectively. Tick DNA/RNA quality was assessed via amplification of the *I*. *ricinus* ITS2 and 16S rRNA fragments respectively as described [48,51].

### High-throughput screening of bacterial and parasitic tick-borne pathogens

The BioMark real-time PCR system (Fluidigm, USA) was used for high-throughput microfluidic real-time PCR for the most common bacterial and parasitic species of tick-borne pathogens known to circulate in Europe, or that might emerge in Europe. Among them, we were able to detect seven species belonging to the Lyme disease spirochete group (*Borrelia burgdorferi* sensu lato): *B*. *burgdorferi* sensu stricto, *B*. *afzelii*, *B*. *garinii*, B. *spielmanii*, *B*. *valaisiana*, *B*. *lusitaniae*, and *B*. *bissettii*. We were also able to detect one species belonging to the *Borrelia* recurrent fever group recently detected in France: *B*. *miyamotoi*. In addition to *Borrelia* species, we were able to detect DNA from five species of *Anaplasma* (i.e. *A*. *phagocytophilum*, *A*. *platys*, *A*. *marginale*, *A*. *ovis*, *A*. *centrale*), *Candidatus* Neoehrlichia mikurensis, *Ehrlichia ruminantium*, *E*. *canis*, *E*. *chaffeensis*. We were also able to detect all Rickettsial species from the spotted fever group using specific primers and probes, *R*. *conorii*, *R*. *slovaca*, *R*. *massiliae*, *R*. *helvetica*. Among *Bartonella* species, we were able to detect *B*. *henselae*, as well as ten species of *Babesia* (*B*. *divergens*, *B*. *microti*, *B*. *caballi*, *B*. *canis*, *B*. *vogeli*, *B*. *venatorum*, *B*. *bovis*, *B*. *bigemina*, *B*. *major*, *B*. *ovis*). Finally, we were able to detect two species of *Theileria* parasite: *T*. *equi* and *T*. *annulata*.

Briefly, a DNA pre-amplification step was performed in a final volume of 5 μL containing 2.5 μL TaqMan PreAmp Master Mix (2X), 1.2 μL of the pooled primer mix (0.2X) and 1.3 μL of tick DNA, with one cycle at 95°C for 10 min, 14 cycles at 95°C for 15 s and 4 min at 60°C. Following pre-amplification, qPCRs were performed using FAM- and black hole quencher (BHQ1)-labeled TaqMan probes [48] with TaqMan Gene Expression Master Mix in accordance with manufacturer’s instructions (Applied Biosystems, France). Thermal cycling conditions were as follows: 95°C for 5 min, 45 cycles at 95°C for 10 s, 60°C for 15 s, and 40°C for 10 s. Data were acquired on the BioMark Real-Time PCR system and analyzed using the Fluidigm Real-Time PCR Analysis software to obtain crossing point (CP) values. Assays were performed in duplicate and two negative water controls were included per chip.

### Reverse transcription real-time PCR for TBEV

RNA samples were screened for TBEV by rRT-PCR targeting a 3’ non-coding region of the TBEV genome using specific primers and probes [51]. rRT-PCR Taqman assays were performed in a final volume of 20 μL using the LightCycler 480 RNA Master Hydrolysis Probes Master Mix (Roche Applied Science, Germany) at 1 X final concentration, with 0.5 μM specific primers and 0.25 μM probes, 3.25 mM manganese acetate [Mn(OAc)_2_] and 2 μL RNA. Positive and negative (water) controls were included in each run. rRT-PCR thermal cycling conditions were as follows: 63°C for 3 min, 95°C for 30 s, 45 cycles at 95°C for 10 s then 60°C for 30 s, followed by cooling at 40°C for 10 s.

### Validation of *B*. *henselae* prevalence by conventional PCR and sequencing

Conventional PCR using primers targeting the *Bartonella* spp. *gltA* gene were used to confirm the presence of *B*. *henselae* DNA in tick samples. Amplicons were sequenced by Eurofins MWG Operon (Germany), and then assembled using BioEdit software (Ibis Biosciences, Carlsbad). An online Blast (National Center for Biotechnology Information) was used to compare results with published sequences listed in GenBank.

### Diagnostic PCR of bacterial symbionts

PCR amplification of bacterial 16S rRNA-encoding *rrs* genes was performed using 2 μL of tick DNA template in 25 μL of reaction mixture containing 5X buffer (New England Biolabs), 40 μM of each deoxynucleoside triphosphate, 0.2 μM of each pA and pH primer (primer details in Table 1), 0.7 U of Q5 High-Fidelity DNA polymerase (New England Biolabs), 1X Q5 high GC enhancer (New England Biolabs) and BSA (0.2 mg mL^-1^). Nested PCR was then used to screen for the presence of *Wolbachia*, *Spiroplasma*, *Acinetobacter*, and *Midichloria* DNA in 2 μL positive *rrs* PCR products. Reactions (25 μL) containing 1X polymerase reaction buffer (Invitrogen, France), 1.5 mM MgCl_2_, 40 μM of each deoxynucleoside triphosphate, 0.2 μM of each primer pair (Table 1) and 0.5 U of Taq DNA polymerase (Invitrogen) were carried out in a T-Gradient Thermocycler (Biometra, France). Each bacterial genus was amplified as previously described [52–55]. For each set of PCR reactions, bacterial DNA extracts from reference strains were used as positive controls. Amplified DNA fragments were separated by electrophoresis through 1% agarose gels stained with ethidium bromide and visualized under ultraviolet illumination.

**Table 1:** Primers and PCR conditions used to amplify the *rrs* gene of symbionts.

Organism	Primer name	Primer sequence (5’– 3’)	Amplicon size (bp) /Tm (°C)	References
Eubacteria	pA	5’—AGAGTTTGATCCTGGCTCAG—3’	1500 / 55	[84]
	pH	5’—AAGGAGGTGATCCAGCCGCA—3’		
*Acinetobacter*	Acin1	5’- ACTTTAAGCGAGGAGGAGGCT—3’	426 / 58	[85]
	Ac	5’—GCGCCACTAAAGCCTCAAAGGCC—3’		
*Spiroplasma*	16STF1	5’—GGTCTTCGGATTGTAAAGGTCTG—3’	964 / 55	[53]
	16STR1	5’—GGTGTGTACAAGACCCGAGAA- 3’		
*Wolbachia*	199F	5’- TTGTAGCCTGCTATGGTATAACT—3’	864 / 52	[52]
	1994R	5’—GAATAGGTATGATTTTCATGT—3’		
*Midichloria mitochondrii*	Midi-F	5’—GTACATGGGAATCTACCTTGC—3’	1100 / 56	[54]
	Midi-R	5’—CAGGTCGCCCTATTGCTTCTTT—3’		

### Detection of associations between pathogens and symbionts in ticks

The statistical likelihood of all possible combinations of pathogens and/or symbionts detectable in this study was analyzed via an association screening approach as described previously [56]. Briefly, the association screening approach comprises a statistical test based on a simulated theoretical distribution of a statistic and its associated confidence interval, under the null hypothesis H0, that pathogen associations are random. The occurrence (i.e. counts) for all possible combinations was theoretically simulated for each pathogen combination, and each combination was unique. The ‘*envelope*’ function from the ‘boot’ package in R software was used to estimate the 95% confidence envelope for the combination count distribution profile, simultaneously including all infection patterns. A global test based on the 95% confidence envelope was initially performed. When H0 was rejected, tests based on the number of possible pathogen combination confidence intervals were performed. Because of the large number of bacterial species that result in an excessive number of combinations compared to the number of ticks, we split the association analyses into three parts: (i) the first concerned associations between the *Borrelia burgdorferi* sl group (comprising *B*. *burgdorferi* s.s., *B*. *garinii*, *B*. *afzelii*, *B*. *valaisiana*, *B*. *spielmanii*) and the six other pathogens (*B*. *miyamotoi*, *A*. *phagocytophilum*, *N*. *mikurensis*, *R*. *helvetica*, *B*. *henselae* and *B*. *divergens*); (ii) the second related to associations among the *Borrelia* sp. group of the six *Borrelia* species; (iii) and the third analyzed symbiont and pathogen associations, for which we analyzed possible interactions between all pathogens (the *Borrelia burgdorferi* s.l. group analyzed as a whole) and each of the different symbionts.

### Environmental drivers of infection

We investigated the effects of environmentally-linked variables (habitat and locality) on the local prevalence of microorganisms (either pathogens or symbionts) within tick populations. Statistical logistic regressions were performed with the R statistical platform using the package MuMIn v.1.7.2 and lme4, with prevalence as the dependent variable, and habitat (forest vs. hedges), and sampling site (nested within habitat) as fixed variables. Model selection was performed using Akaike’s Information Criterion (AIC) [57]. The model with the lowest AIC value was viewed as the most parsimonious, i.e. the model which explains the majority of variance with the fewest parameters [57].

## Results

### Pathogen prevalence according to habitat

**Fig 2:**
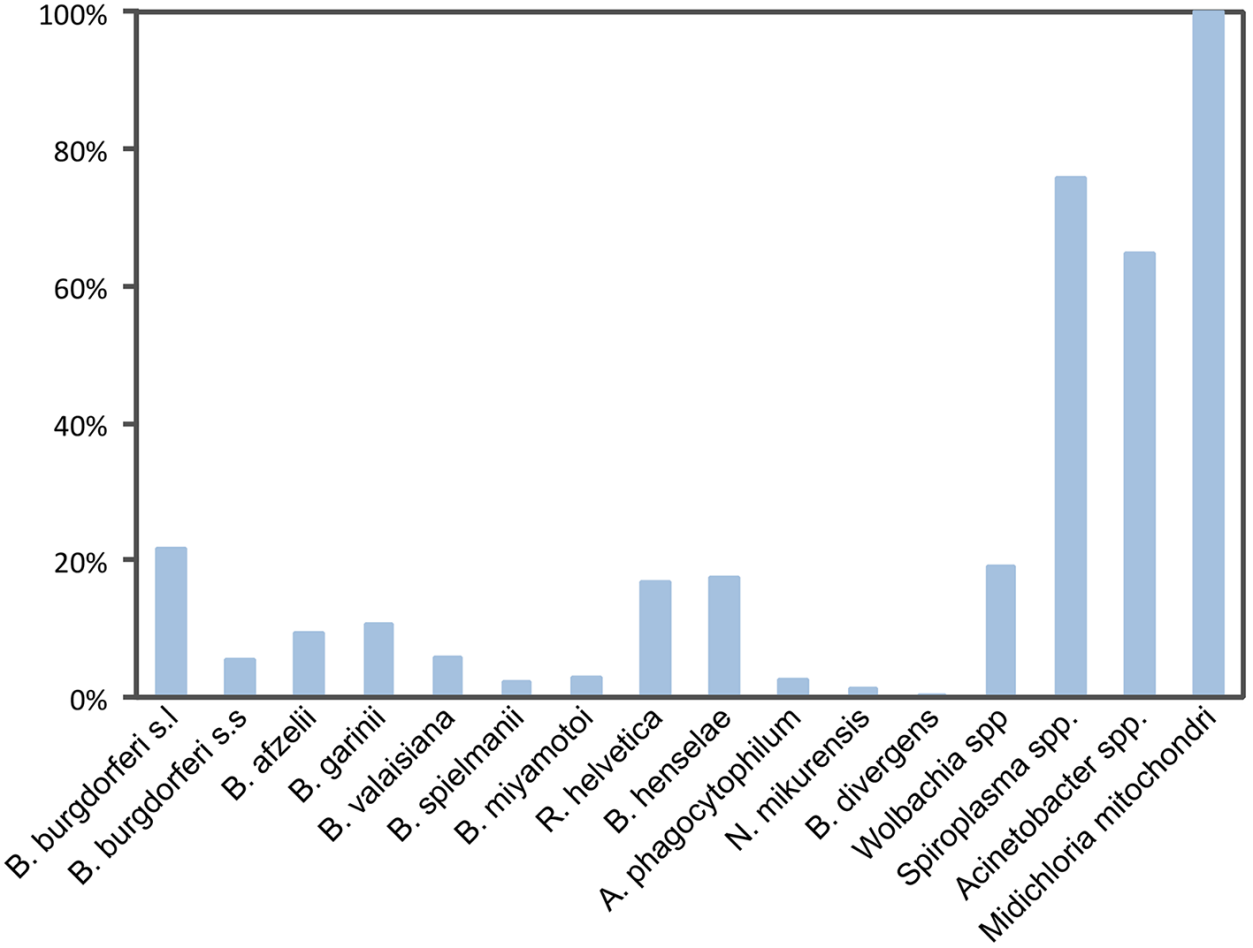
DNA prevalence of the most common tick-borne pathogens and putative symbionts in *I*. *ricinus* ticks.

In this study, we analyzed *I*. *ricinus* for the presence of bacterial or parasitic DNA, including the most common tick-borne pathogens circulating in Europe (Fig 2), as well as TBEV RNA. Among the 267 individually analyzed female ticks, almost half (45%) were infected by at least one pathogen. Of these, the most prevalent nucleic acids belonging to pathogenic agent were those affiliating to Lyme disease spirochetes [21.7% in total; including *B*. *burgdorferi* sensu stricto (5.6%), *B*. *afzelii* (9.4%), *B*. *garinii* (10.8%), *B*. *valaisiana* (6.0%), and *B*. *spielmanii* (2.2%)]. The next most prevalent nucleic acids corresponded to *Bartonella henselae* (17.6%) and *Rickettsia* of the spotted fever group (16.8%), which were mostly *Rickettsia helvetica*. Due to the unexpectedly high prevalence of *B*. *henselae*, all ticks that returned positive qPCR results were also tested by conventional PCR for the *Bartonella* g*ltA* gene. All samples gave positive PCR amplicons, which after sequencing demonstrated that all samples shared 100% identity with *B*. *henselae* (Houston I strain), confirming that 17.6% of ticks carried *B*. *henselae* DNA.

Besides these highly prevalent pathogens, other pathogens with lower prevalences were also detected: *Borrelia miyamotoi* (3.0%), *Anaplasma phagocytophilum* (2.6%), Candidatus *Neoehrlichia mikurensis* (1.4%), and *Babesia divergens* (0.37%). We failed to detect TBEV, other *Borrelia* species *(B*. *lusitaniae* and *B*. *bissettii)*, other *Babesia* species (B. *microti*, *B*. *caballi*, *B*. *canis*, *B*. *vogeli*, *B*. *venatorum*, *B*. *bovis*, *B*. *bigemina*, *B*. *major* and *B*. *ovis*), *Theileria* species (*T*. *equi*, *T*. *annulata*), other *Rickettsia* species (R. conorii, R. slovaka, R. massiliae), *Ehrlichia* species (E. ruminantium, E. canis, E. chaffeensis), and other *Anaplasma* species (*A*. *phagocytophilum*, *A*. *platys*, *A*. *marginale*, *A*. *ovis*, *A*. *centrale*).

Statistical models indicated significant variation in the prevalence of *B*. *burgdorferi* sensu lato, *B*. *henselae*, and *R*. *helvetica* amongst sampling sites (Table 2). For the other microorganisms, none of the variables were retained in the most parsimonious model, thus suggesting that their prevalence was not clearly related to locality or the type of habitat.

**Table 2:** Prevalence (%) of the microorganisms detected in ticks according to localities and landscape. Infection indicates the % of ticks infected by at least one pathogen. Coinfection represents the % of ticks infected by at least two pathogens. M ± SD is mean ± standard deviation.

Locality	Cassine	Croixbois	Elan	Hargnies	Renwez	Woiries		Briquenay	Cliron	Sauville		
Landscape	Forest	Forest	Forest	Forest	Forest	Forest	Forest (M±SD)	Hedge	Hedge	Hedge	Hedge (M ± SD)	TOTAL (M ± SD)
***Borrelia burgdorferi***	0.0	0.0	3.3	2.7	12.0	18.5	6.1 ± 7.5	3.1	0.0	3.6	2.2 ± 1.9	4.8 ± 6.3
***Borrelia garinii***	3.8	3.6	6.7	5.4	24.0	18.5	10.3 ± 8.7	6.3	0.0	14.3	6.8 ± 7.2	9.2 ± 8.0
***Borrelia afzelii***	7.7	3.6	3.3	5.4	22.0	11.1	8.9 ± 7.1	9.4	0.0	7.1	5.5 ± 4.9	7.7 ± 6.3
***Borrelia valaisiana***	3.8	0.0	6.7	5.4	6.0	3.7	4.3 ± 2.4	9.4	0.0	14.3	7.9 ± 7.3	5.5 ± 4.5
***Borreliaspielmanii***	0.0	0.0	0.0	5.4	4.0	7.4	2.8 ± 3.3	0.0	0.0	0.0	0.0 ± 0.0	1.9 ± 2.9
***Borrelia miyamotoi***	0.0	0.0	6.7	2.7	4.0	0.0	2.2 ± 2.8	6.3	0.0	3.6	3.3 ± 3.1	2.6 ± 2.7
***Anaplasma phagocytophilum***	0.0	7.1	0.0	2.7	4.0	0.0	2.3 ± 2.9	0.0	0.0	7.1	2.4 ± 4.1	2.3 ± 3.1
***Neoehrlichia mikurensis***	0.0	0.0	3.3	0.0	4.0	3.7	1.8 ± 2.0	0.0	0.0	0.0	0.0 ± 0.0	1.2 ± 1.8
***Ricketssia helvetica***	11.5	28.6	30.0	0.0	12.0	7.4	14.9 ± 11.9	31.3	0.0	25.0	18.8 ± 16.5	16.2 ± 12.7
***Bartonella henselae***	3.8	0.0	33.3	10.8	36.0	14.8	16.5 ± 15.0	0.0	11.1	32.1	14.4 ± 16.3	15.8 ± 14.5
***Babesia divergens***	0.0	0.0	0.0	0.0	2.0	0.0	0.3 ± 0.8	0.0	0.0	0.0	0.0 ± 0.0	0.2 ± 0.7
**Infection**	19.2	28.6	56.7	27.0	70.0	40.7	40.4 ± 19.5	43.8	11.1	67.9	40.9 ± 28.5	40.6 ± 21.0
**Coinfection**	7.7	7.1	30.0	5.4	34.0	18.5	17.1 ± 12.5	12.5	0.0	28.6	13.7 ± 14.3	16.0 ± 12.3
***Wolbachia sp*.**	0.0	35.7	11.5	11.1	0.0	70.4	21.5 ± 27.3	26.7	0.0	20.0	15.6 ± 13.9	19.5 ± 22.8
***Spiroplasma sp*.**	76.9	67.9	65.4	91.7	83.3	44.4	71.6 ± 16.5	80.0	100.0	76.0	85.3 ±12.9	76.2 ± 16.1
***Acinetobacter sp*.**	96.2	89.3	100	27.8	52.1	18.5	64.0 ± 36.0	100.0	44.4	60.0	68.1 ± 28.7	65.4 ± 32.0
***Midichloria mitochondrii***	100	100	100	100	100	100	100 ± 0.0	100	100	100	100 ± 0.0	100 ± 0.0

### Co-infections and associations between pathogens

**Fig 3:**
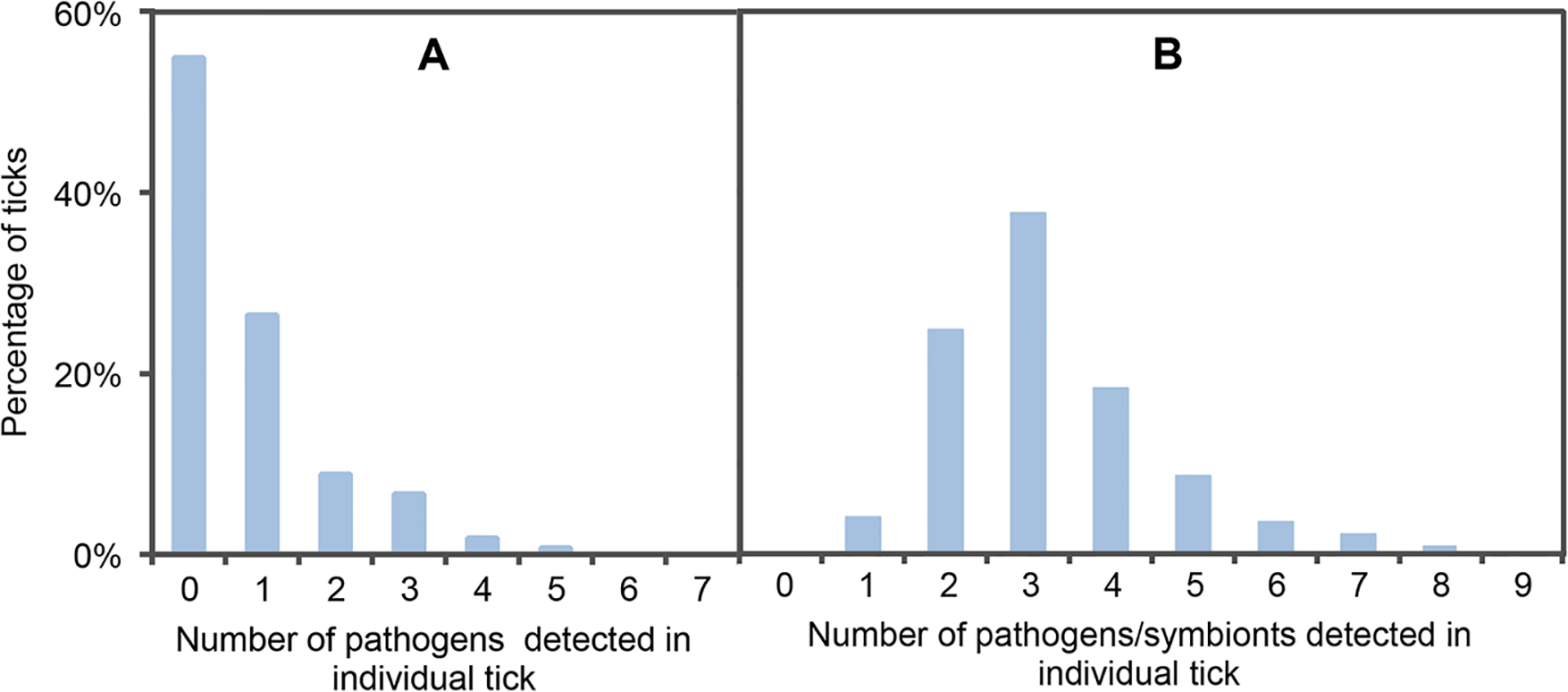
**Co-infections levels for pathogens (A) or for pathogens and symbionts (B).**

Among all infected ticks (120/267, 45% of all collected ticks) half were found to be co-infected (54 co-infected ticks out of 120 infected ticks) (Fig 3A): 9% carried DNA from two pathogen species, 6.7% carried DNA from three pathogens, 1.9% carried DNA from four pathogens, and 0.75% carried DNA from five different pathogens. When performing statistical analyses of bacterial associations, where only the five *Borrelia* species were included, five combinations were significant (p-values: <10^−3^): two co-infection patterns were found to be significantly over-represented, one between *Borrelia burgdorferi* sensu stricto, *B*. *garinii*, *B*. *afzelii*, and *B*. *spielmanii*, and the other between *Borrelia garinii* and *B*. *afzelii*; while three combinations were significantly under-represented, these included two single infections (*Borrelia garinii* and *B*. *afzelii*), and combinations concerning non-infected individuals. When analyzing all pathogens together, and when the *Borrelia burgdorferi* sensu lato group was analyzed as a single pathogen, no combinations were found to be significant (Table 3).

**Table 3:** Analysis of significant *Borrelia* combinations in ticks.

*Borrelia* combinations*						N [CI] **	P-value
Bbss	Bg	Ba	Bv	Bs	Bm		
1	1	1	0	0	0	3 [0–1]	<10^−3^
0	1	1	0	0	0	11 [0–8]	<10^−3^
0	1	0	0	0	0	4 [11–36]	<10^−3^
0	0	1	0	0	0	5 [8–33]	<10^−3^
0	0	0	0	0	0	210 [158–205]	<10^−3^

*In each combination “0” or “1”correspond to “Absence” or “Presence” of one of six *Borrelia* species: Bbss = *B*. *burgdorferi* sensu stricto, Bg = *B*. *garinii*, Ba = *B*. *afzelii*, Bv = *B*. *valaisiana*, Bs = *B*. *spielmanii* and Bm = *B*. *miyamotoi*.

** N = number of combinations observed, CI = confidence interval of the statistical envelope

### Interactions between symbionts and pathogens

We were able to estimate the prevalence of four known or suspected symbionts associated with ticks, i.e., *Wolbachia*, *Spiroplasma*, *Acinetobacter*, and *Midichloria mitochondrii*. Tick samples were only utilized in prevalence analysis if the *rrs* gene could be successfully amplified prior to specific amplification. From a total of 255 analyzed tick specimens, *M*. *mitochondrii* DNA was detected in all ticks (100% prevalent), *Spiroplasma* spp. DNA was found in 75.7% of ticks, *Acinetobacter* spp. in 64.7% of ticks, and *Wolbachia* DNA in 19.2% of ticks (Fig 2). Analysis of the 823 bp PCR products identified several different *Wolbachia wsp* genes which aligned with KT285474, KT285475, KT285476, KT285477, and KT285478 sequences. PCR products shared 99% identity with *Wolbachia wsp* sequences previously reported as associated with insects and parasitoid Hymenoptera: the parasitoid *Nasonia vitripennis* (M84686.1), the parasitoid *Pteromalus puparum* (EU827689.1), the birch catkin bug *Kleidocerys resedea* (JQ726770.1) [22], the pigeon fly *Pseudolynchia canarienis* (GQ167624.1), and *Colpodes buchanani* (GU236928.1). Accordingly, due to these high rates of infection, the overall co-infection rate also increased when symbionts were added to the pathogen analyses; since all ticks were found to be infected by at least one microorganism, and some ticks were carrying up to eight microorganisms each (Fig 3B).

Statistical models indicated significant variation in the prevalence of *Wolbachia* sp., *Spiroplasma* sp., and *Acinebacter* sp. amongst sampling sites (Table 2). When assessing possible interactions between symbionts and pathogens, no combinations were found to be significant.

## Discussion

Using powerful high-throughput epidemiological tools we achieved a comprehensive overview of the epidemiological situation of tick-borne pathogens circulating in ticks from the French Ardennes. The most significant findings were; 1) the high overall levels of pathogen infection, since half of all ticks (120 out of 267) carried at least one pathogen and 2) frequency of co-infection, as half of the infected ticks (54 out of 120) were infected by at least two pathogens. Both novel results may have important implications in terms of public health issues [19–25,29].

First of all, co-infections with different tick-borne pathogens in both humans and animals are commonly reported [20,22–25,58,59]. For instance, in the USA it was shown that 11% of Lyme disease patients in southern New England also experienced concurrent babesiosis [59], and 13% of Lyme disease patients in Wisconsin also contracted human granulocytic anaplasmosis [60]. Of 310 North American patients suspected to be infected with tick-borne disease and screened for Lyme disease, HGA, and Babesiosis, 117 had single infections, 75 had co-infections, and 4 individuals were positive for Lyme disease, HGA, and Babesiosis [58].

Tick-borne co-infections can have enormous impact on the diagnostic methods utilized. For instance, the diagnosis of tick-borne disease in France is almost uniquely limited to Lyme Borreliosis. The detection of many different pathogenic species in ticks indicates that diagnostic tools must match the range of pathogens carried by ticks from every specific geographical area. Another important public health issue is that co-infections may enhance disease severity, or alter typical symptoms, thus impeding diagnosis [59]. For instance, co-infected Lyme disease patients harbored more influenza-like symptoms than those with Lyme disease alone [59]. In the case of concurrent babesiosis and Lyme disease, co-infected patients experienced a greater number of symptoms for a longer duration than those with Lyme disease alone [59]. And finally, co-infections can affect tick-borne disease treatment, as antibiotic therapies prescribed for Lyme disease are not efficient against parasitic or viral diseases.

In this context, adopting a perspective that takes co-infection into account may help to identify more causes of tick-borne disease, and thus aid in the development of adapted diagnostic tools and treatments [29].

Despite the importance of nymphs in terms of pathogen transmission, we chose to investigate co-infection in adult ticks for two main reasons: firstly, because they feed once more than nymphs and are more likely to be (co)-infected; secondly, even though nymphs are thought to be more relevant to public health (bites may remain undetected due to their small size), adults may actually be more important than previously thought. Contrary to popular belief, female adult ticks are capable of biting humans (although probably in lower proportions than nymphs) [5–8], and although female ticks are likely more easily noticed and removed before the end of their blood meal due to their larger size, perhaps reducing pathogen transmission time [4], transmission might start before the tick bite is noticed and adult tick removal. Combined with the fact that adults are more likely to be co-infected, and that co-infections in humans are difficult to diagnose, studying adult tick co-infection appears highly relevant. In any case, similar future studies should be performed on nymphs to assess co-infection levels in this highly relevant tick stage.

The most prevalent tick pathogens were those linked to Lyme disease, which is not entirely surprising due to the region’s close proximity to Alsace, the most important French territory endemic for Lyme disease. Of the five French *Borrelia* species responsible for Lyme disease, *B*. *garinii* and *B*. *afzelii* were the most prevalent in ticks, with Lyme borreliosis spirochete species detected in 21.7% of ticks. This infection rate reflects previously published results, with prevalences ranging from 6% in Western France, to 32% in forests around Paris [61], and 36% in Alsace [62]. In addition to Lyme Borreliosis spirochetes, we also identified *Borrelia miyamotoi* in 3% of ticks. Similar to Lyme borreliosis etiological agents, this pathogenic species is transmitted by the same *Ixodes* tick species, but causes different clinical manifestations (relapsing fever, without erythema migrans). Up until now no human cases have been reported in France, but our data and the recent case of human infection described in the Netherlands [63] suggest that surveillance urgently needs to be improved.

Interestingly, the most prevalent pathogens after Lyme spirochetes were *B*. *henselae* (17.6%) and *R*. *helvetica* (16.8%). Over recent years many reports have identified *B*. *henselae* DNA in ticks and in patients bitten by ticks [64,65], and *I*. *ricinus* has also been identified as a competent vector for *B*. *henselae* [66]. Notwithstanding these findings, the role of ticks as a vector of *B*. *henselae* is still heartily debated, where the faction “against” argues that if transmission has occurred, it may only be anecdotal. Prevalence of *B*. *henselae* DNA in ticks varies greatly between countries, with no positive detection in the Netherlands, to a confirmed significant prevalence in ticks from Germany [64]. Variation may also occur at a national level. For example, *B*. *henselae* prevalence in ticks from France varied from 1% in ticks collected near Paris [48] to 38% in some specific regions of Alsace [64]. These significant differences could be explained by the differential presence of *B*. *henselae* reservoirs in the studied areas. For *B*. *henselae*, the most common reservoir animals are *Felidae*, including wild cats [67,68]. Interestingly, wild felids were known to be present in the Ardennes forest from which ticks were collected, and thus may serve as the actual reservoir for this bacterium. In addition to felids, it has been frequently debated whether rodents could also play a role as a possible *B*. *henselae* reservoir. One study isolated *B*. *henselae* from *Apodemus sylvaticus* wood mice, which also happens to be a common *Ixodes* larval host [69]. In addition to tick survey, we have also conducted the identification of *Bartonella* species in small mammals collected in France (in a forest located in Southeastern Paris) but did not detect any *B*. *henselae* despite high prevalence of other *Bartonella* species confirming that rodents are unlikely to be infected by *B*. *henselae* [70].

*R*. *helvetica* has previously been described as prevalent in ticks throughout Europe, and this finding was replicated in our study [71,72]. It is still unknown whether an animal reservoir exists for this bacterium, and thus the tick is thought to be the natural reservoir. Its pathogenicity in humans also remains unclear. For instance, *R*. *helvetica* has been incriminated in the development of fatal perimyocarditis, however isolation of the bacterium from a patient is still required to definitively confirm its pathogenic status as a cause of perimyocarditis [73].

Following these highly prevalent pathogens, we detected much lower prevalence levels for *A*. *phagocytophilum* and the possible tick-borne pathogen *Candidatus* Neoehrlichia mikurensis. Both are known to be pathogenic for humans and/or animals. However clinical symptoms caused by these agents are not pathognomonic, and could be indicative of other pathogens. Because public health professionals may be unaware of these types of diseases, multiple unreported cases may exist. Monitoring of these bacteria must be enhanced to the level of other tick-borne pathogens.

Interestingly, we detected *Babesia* species (*B*. *divergens*) at very low prevalence rates (0.3%), which might be explained by the absence of its natural bovine host in the collected area.

In this study, all of these pathogens were found to co-exist within individual ticks (up to five species of pathogens in the same tick). When considering possible interactions between pathogens, the association results are suggestive of a strong association between *Borrelia garinii and Borrelia afzelii* in ticks. *Borrelia garinii* or *Borrelia afzelii* are implicated in four of the five significant combinations: they are detected together more frequently than for any other two combinations (Table 3), as a result, they are less frequently detected alone. *B*. *garinii* and *B*. *afzelii* are known to have different reservoir hosts ([74,75], birds and rodents respectively). Female ticks can contract infection at both the larval and nymph stages. Thus, the detected association between *B*. *garinii* and *B*. *afzelii* could result from: (i) immature stages having fed on both birds and rodents (ii) ticks contracting *Borrelia* species other than the *Borrelia* which infects their host during co-feeding, (iii) and/or biological interactions between the two *Borrelia* species that facilitate co-infection. Furthermore, our findings suggest a possible role for the combination of *Borrelia garinii* and *B*. *afzelii* together, which favors the establishment of bacteremia by *Borrelia burgdorfe*ri s.s. and *Borrelia spielmanii*. In addition, given the observed prevalence of the five *Borrelia* species, a surprising number of ticks were actually free from *Borrelia*, likely caused by the under-representation of infections with only *B*. *garinii* or *B*. *afzelii*.

High prevalence rates may indicate intimate symbiotic relationships between a given bacterium and its host. For at least three species, *M*. *mitochondrii* (100%), *Spiroplasma* (75.7%), and *Acinetobacter* (64.7%), such high prevalences were actually observed, thus conferring probable symbiont status to these *I*. *ricinus* bacteria. Previous studies have demonstrated *M*. *mitochondrii* presence in 100% of *I*. *ricinus* females within a geographical locality [40,41]. Interestingly, it was demonstrated that *M*. *mitochondrii* can reside in tick salivary glands [76]. These authors suggested that *M*. *mitochondrii* could modulate the host’s immune response via *I*. *ricinus* saliva, which is important for both the success of the tick blood meal and for initiating infection by tick-vectored pathogens. Despite the importance of *I*. *ricinus* to both human and veterinary medicine, the full consequences of *M*. *mitochondrii* presence on the biology of its host arthropod remain unknown.

The genus *Spiroplasma* encompasses diverse poorly characterized species, including commensal, mutualistic, and pathogenic organisms [77,78]. While many categories of insects and arachnids harbor these bacteria, little information is available on the prevalence and distribution of *Spiroplasma* in natural tick populations. A recent study showed that *Spiroplasma* and *Coxiella* dominated bacterial microbiota in tick salivary glands, accounting for more than 90% of the bacterial community in *Ixodes ovatus* [79]. These results are in accordance with our findings and again suggest a symbiotic association between this bacterium and ticks.

To our knowledge, this is the first study estimating *Acinetobacter* prevalence in ticks. The relatively high prevalence described here (64.7%), poses further questions as to how *I*. *ricinus* females acquire and transmit the bacterium. *Acinetobacter* are often found associated with hematophagous arthropods [55,80] and its role in insect biology has only been demonstrated in the fly *Stomoxys calcitrans*, which requires these bacteria to ensure complete larval development [81].

Surprisingly, we found an overall *Wolbachia* infection frequency of 19.2% in female *I*. *ricinus*. This indeed contrasts with previous infection prevalence values of 1.0% in French adult *I*. *ricinus* ticks [61], or with other European prevalence studies, where 0.9% of adult *I*. *ricinus* ticks were infected in Southern Germany [82], and where it was not detected in ticks from the west coast of Norway [83]. It was recently demonstrated that *Wolbachia* in ticks is actually due to the seemingly cryptic interaction of the *I*. *hookeri* wasp [39]. This hymenopteran endoparasitoid mostly parasites nymphs, and whose existence only becomes visible after tick engorgement. The adult tick’s innate immune response against parasitoid eggs likely explains the lower *Wolbachia* infection rate in adult ticks [39]. More efforts are needed to fully understand the apparently contradictory higher infection prevalence found in this study.

Overall, statistical association analysis between symbionts and pathogens did not reveal any significant associations. This might indicate that the presence of symbionts does not affect the presence of pathogens. However, even though no synergistic or antagonistic associations were observed, this does not exclude the possibility of more subtle functional interactions between commensal microbiota with potential impact on pathogen acquisition, transmission, and virulence that are not currently revealed by our statistical tests and the limited number of ticks analyzed. Another limitation of our study is the use of adult stage only. Indeed, nymphs are also a very important stage in terms of public health and a better knowledge of the importance of co-infection as well as the possible interaction between pathogens and symbionts in nymphs will be of great interest.

In conclusion, our study reveals high pathogen co-infection rates in adult ticks. Even though additional larger studies on nymph co-infection are yet to be completed, this primary result raises questions about the possibilities of co-transmission of these agents to humans (or animals), their prevalence, the effects of these co-infections on the severity of symptoms, the efficacy of treatments, and how to develop new diagnostic tests better adapted to tick-borne diseases. We also demonstrated high prevalence of symbionts co-existing with pathogens. It is now important to address and comprehend their effect on pathogen transmission and/or tick biology. Improved knowledge on the functional and evolutionary consequences of tick microbiota-pathogen interactions should boost the development of new alternative strategies for the control of ticks and tick-borne pathogens.
